# Epilepsia partialis continua may be a rare electroclinical feature of anti-Hu encephalitis: a pediatric case report

**DOI:** 10.3389/fped.2026.1775494

**Published:** 2026-04-22

**Authors:** Jialei Chen, Xingyou Wang, Jing Zhang, Yanjuan Wang, Wenguang Hu

**Affiliations:** 1 Pediatric Neurology Department, Chengdu Women’s and Children’s Central Hospital, School of Medicine, University of Electronic Science and Technology of China, Chengdu, China; 2 Chongqing Medical University-University of Leicester Joint Institute, Chongqing, China

**Keywords:** anti-Hu encephalitis, epilepsia partialis continua, immunotherapy, neuroblastoma, pediatrics

## Abstract

**Background:**

Paraneoplastic neurologic syndromes with anti-Hu antibodies refer to a spectrum of neurologic disorders. Rare cases of extra-limbic encephalitis presenting with seizures have also been reported. However, these seizures rarely manifest as epilepsia partialis continua (EPC). We report an anti-Hu encephalitis pediatric case with EPC.

**Case description:**

A 3-year-and-4-month-old male presented with EPC after neuroblastoma resection for 6 months. Neuronal antibody testing revealed highly positive anti-Hu antibody in both serum and cerebrospinal fluid. Anti-Hu encephalitis with EPC was diagnosed. Initial immunotherapy including intravenous immunoglobulin and methylprednisolone had a transient effect, and the symptom recurred 1 month later. The EPC still existed even with the treatment of prednisone, mycophenolate mofetil, oxcarbazepine, valproic acid, and clonazepam.

**Conclusion:**

EPC may be a rare but underrecognized electroclinical feature of anti-Hu encephalitis. It is important to consider anti-Hu encephalitis in patients presenting with new-onset EPC. The heterogeneity in clinical symptoms, EEG and imaging manifestations may indicate the existence of complex and diverse mechanisms. The prognosis of this disease is speculated to be poor. Antiseizure medications and surgery often fail to control the progression of the disease. Immunotherapy may benefit some patients.

## 1 Introduction

Anti-Hu is the most frequent antibody in paraneoplastic neurologic syndromes. According to the updated diagnostic criteria for paraneoplastic neurologic syndromes, anti-Hu antibodies are considered high-risk antibodies strongly associated with an underlying malignancy (1). More than 85% patients with anti-Hu antibodies have small cell lung cancer (SCLC) in adult and anti-Hu antibodies are associated with neuroblastoma or ganglioneuroblastoma in children (1–3). Paraneoplastic neurologic syndromes with anti-Hu antibodies refer to a spectrum of neurologic disorders, including limbic encephalitis, peripheral neuropathy, cerebellar ataxia and autonomic dysfunction. Seizures most commonly occur in the setting of limbic encephalitis, although rare cases of extra-limbic encephalitis presenting with seizures have also been reported in the literature (4). However, these seizures rarely manifest as epilepsia partialis continua (EPC).

EPC is a rare type of focal motor seizure characterized by continuous, involuntary muscle contractions in a specific part of the body lasting for several hours to days. Immune-related disease is the most frequent etiology in children, among which approximnately 90% of the causes is Rasmussen syndrome (5). Moreover, rare cases have been reported in anti-Hu encephalitis. Herein, we report an anti-Hu encephalitis pediatric case with EPC and reviewed the clinical characteristics, EEG feature, imaging findings, laboratory data, treatments, and prognosis of anti-Hu encephalitis patients with EPC, to get a better understanding of anti-Hu encephalitis.

## 2 Case presentation

**Figure 1:**
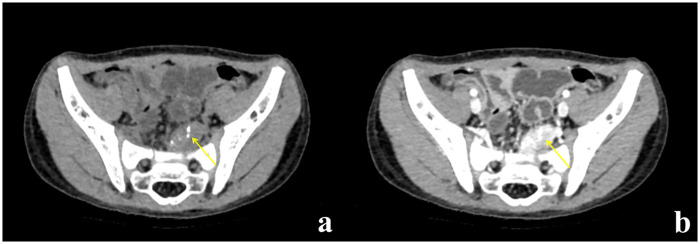
Pelvis CT scan. **(a)** CT scan showed a mass beside the left internal iliac vessel. **(b)** Enhancement could be seen on contrast-enhanced CT scan. The yellow arrow indicated the mass.

A 2-year-and-7-month-old male was the first child of non-consanguineous Chinese parents with no relevant past medical history. His psychomotor development was apparently normal. He presented with ataxic gait for 3 days. Diagnostic workup included cerebrospinal fluid (CSF), brain and spinal cord MRI which were initially unremarkable. Based on clinical features he was diagnosed with cerebellar ataxia. His symptoms completely recovered after intravenous immunoglobulin (IVIG) 2 g/kg and dexamethasone for 5 days. However, the symptoms recurred 2 months later when he was 2-year-and-9-month-old. He was again admitted to the hospital. Repeated CSF analysis, brain and spinal cord MRI had no abnormal findings. In the context of paraneoplastic screening, a chest, abdomen, and pelvis CT scan were performed and a contrast enhancement mass with 24 mm × 14 mm × 30 mm was found beside the left internal iliac vessel (Figure 1). The patient underwent complete resection of the posterior peritoneum mass and pathological analysis confirmed neuroblastoma, differentiating subtypes (N-Myc not amplified, low mitosis-karyorrhexis index). He was started on 4 sessions of chemotherapy (carboplatin, cyclophosphamide, doxorubicin, etoposide). And IVIG (2 g/kg) was used again to attenuate the ataxic symptom. The ataxia improved within 5 months.

The patient developed involuntary focal jerking of the right fingers and arm without alteration of consciousness during both awake and sleep when he was 3-year-and-4-month-old. The jerks of fingers and arm were initially intermittent but became continuous within over the following 2 weeks. There were occasional clonic jerks of the right leg and the right side of the face. Those seizures often lasted more than one hour, occurred 12–16 times per day. Compared with sleep, the seizures were more frequent during wakefulness. No opsoclonus was observed during the course of the disease. Neurological examination at that time showed ataxia, slight paralysis (Medical Research Council grade 4) of the right upper limb and the positive Babinski sign on the right side. His gait was wide-based and the remainder of the examination was normal. The myoclonic jerks of the right fingers and arm were observed.

**Figure 2:**
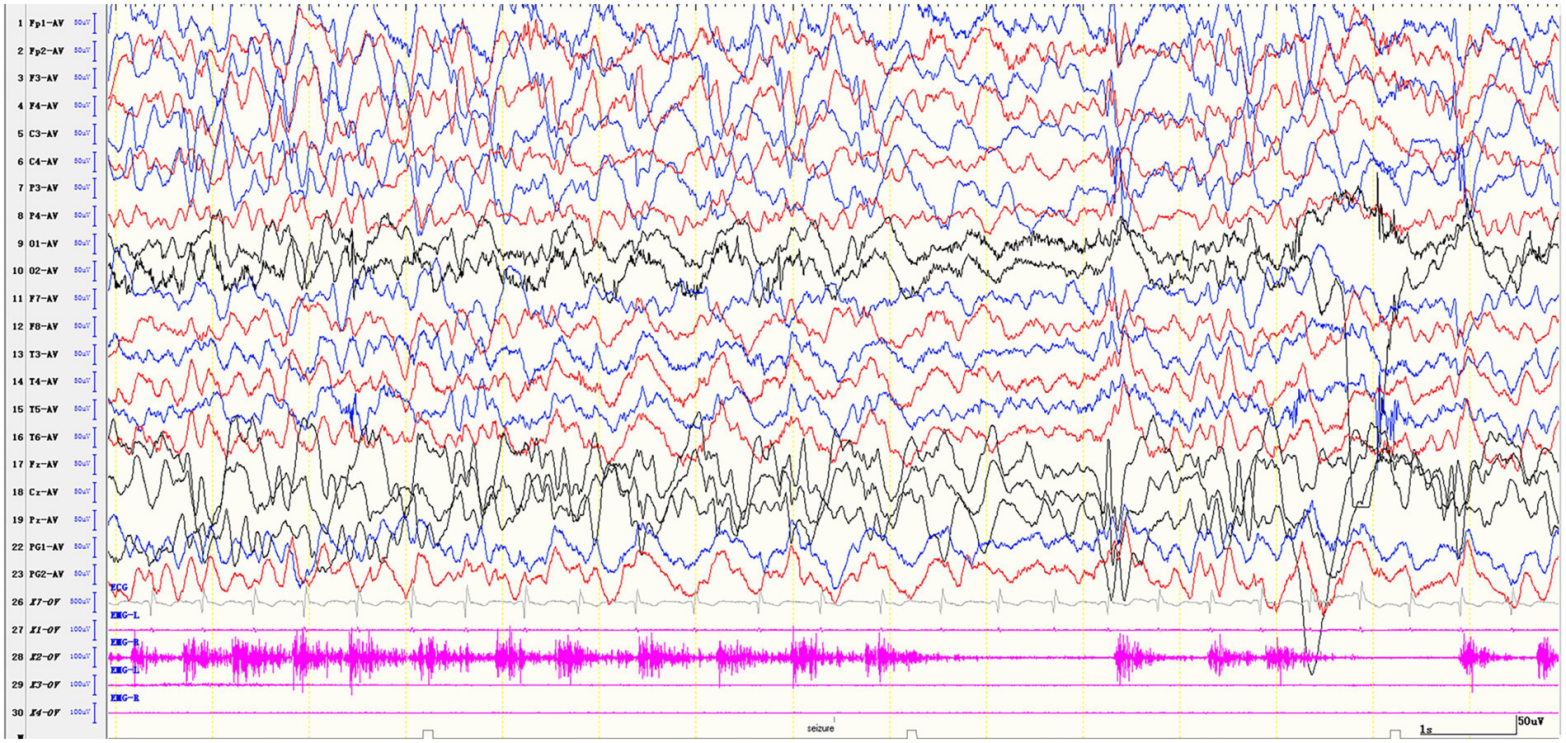
Ictal EEG. Continuing rhythmic sharp-slow wave activities were recorded in the left fronto-central-parietal regions. EMG on the right thenar muscle recorded regular myoclonus. EEG was displayed in an average montage, time base 30 mm/s, LFF 1 Hz, HFF 70 Hz.

**Figure 3:**
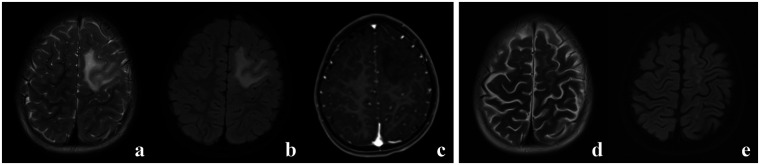
Brain MRI. Hyperintensity signal could been seen on T2-weighted **(a)** and FLAIR **(b)** in the left frontal region without enhancement on T1, plus gadolinium **(c)**. Follow-up MRI performed 3 months after EPC onset showed a significant decrease in the area of hyperintensity on T2-weighted **(d)** and FLAIR **(e)**.

The EEG demonstrated continuing rhythmic sharp-slow wave activities in the left fronto-central-parietal regions, which were synchronous with an observed myoclonus of the right upper limb (Figure 2), suggestive of EPC. Brain MRI revealed hyperintensity signal on T2-weighted and fluid attenuation inversion recovery (FLAIR) in the left frontal region without any mass effect and contrast enhancement (Figures 3a–c). The patient was treated with oxcarbazepine (OXC), but it was ineffective. Considering symptom persistence, a comprehensive work-up was performed. There was no evidence for recurrence and metastasis of his previous cancer on CT, including liver, lymph node and bones. Tumor markers were normal, except for neuron specific enolase which was slightly elevated (40.883 ng/mL). CSF analysis showed 2 white blood cells, normal protein level (150.7 mg/L) and CSF glucose (3.38 mmol/L). Systemic autoimmune studies and common infectious causes of encephalitis were unremarkable. Neuronal antibody testing revealed highly positive anti-Hu antibody at 1:1,000 in serum and 1:100 in CSF but was negative for other anti-neuronal cell-surface and intracellular antibodies. The oligoclonal bands were positive. The patient was treated with IVIG 2 g/kg and pulse steroids (methylprednisolone 20 mg/kg/d for 5 days, followed by oral prednisone at 2 mg/kg/d with slow progressive tapering). After the treatment of immunotherapy for 2 weeks, the EPC disappeared, and the paralysis improved. Nevertheless, 1 month later, the myoclonic jerks of the right extremity recurred. Repeated evaluation of serum antibodies revealed a continued positive anti-Hu antibody. Mycophenolate mofetil (MMF) 600 mg/m^2^/d was started, alongside valproic acid (VPA) 20 mg/kg/d, OXC 40 mg/kg/d, clonazepam (CZP) 0.12 mg/kg/d and prednisone 2 mg/kg/d. Follow-up MRI images of 3 months after EPC onset showed a significant decrease in the area of abnormal signal and no progressive hemiatrophy was observed (Figures 3d,e). The ataxia alleviated and the paralysis of the right upper limb fully recovered, but the EPC still existed as of the last follow-up at the age of 3 years and 10 months.

## 3 Discussion

EPC, although variable definitions exist is generally defined as a syndrome of continuous, localized motor seizures that persist for more than an hour, often over the course of days or even years (6). It may manifest as prolonged repetitive and regular muscle jerks, typically affecting a single body part, commonly the distal muscles of the upper extremities. In the recent International League Against Epilepsy task force report on status epilepticus and operational classification, EPC can be categorized within the spectrum of focal motor status epilepticus (7, 8). The pathophysiology of EPC is complex and not completely understood. It is believed to involve a combination of factors and different etiologies that lead to the generation and maintenance of abnormal electrical activity in the motor cortex. These etiologies can cause an imbalance between excitatory and inhibitory neurotransmission, changes in ion channel function, and alterations in synaptic connectivity. Moreover, the spread of abnormal electrical activity to adjacent cortical and subcortical structures may contribute to the persistent motor symptoms observed in EPC (9). EPC has multiple underlying causes in children, including immune-related disease, focal lesions, metabolic and genetic disease (5). Autoimmune condition is a well-known cause of EPC. It may lead to an inflammation cascade and result in seizures (9).

EPC has been reported in autoimmune encephalitis including anti-NMDA receptor encephalitis and LGI1 antibody encephalitis (9, 10). Moreover, rare cases have been reported in anti-Hu encephalitis. To date, EPC associated to anti-Hu encephalitis has been clearly reported in 9 patients (3, 11–16) and it may be a rare but underrecognized electroclinical feature of anti-Hu encephalitis. Table 1 summarizes the clinical characteristics, EEG features, imaging findings, laboratory data, treatments, and prognosis of previous anti-Hu encephalitis cases with EPC. Among the 9 patients, only 2 were children. And our case is the youngest. Recent reports have further highlighted the heterogeneity of pediatric anti-Hu-associated paraneoplastic syndromes, particularly in association with neuroblastic tumors (17). Consistent with the recent reports, the symptoms of the 9 patients were diverse and not all patients had the typical symptoms of limbic encephalitis such as cognitive dysfunction, behavioral and personality changes. The ictal EEG showed clear synchronous relationship between abnormal discharge and myoclonus in all the patients. However, the electro-clinical-radiological relationships were complex and diverse. The location of discharges on EEG did not exactly correspond to the area of abnormal signal on MRI. The MRI showed multifocal areas of abnormal signal in three cases (12, 13, 15), while one had completely normal imaging (11). Our case had a recurrence of EPC when the area of abnormal signal on MRI significantly decreased. These suggested that apart from structural abnormalities, immune response was the main cause of EPC in anti-Hu encephalitis. How does EPC occur in anti-Hu encephalitis is still unclear. It was speculated that multifocal infiltrates of inflammatory cells lead to the development of multiple foci of neuronal hyperexcitability and that myoclonic jerks might result from an atypical propagation of the neuronal activity along multiple networks (18). Considering the heterogeneity in clinical symptoms, EEG and imaging manifestations in patients of anti-Hu encephalitis with EPC, there may exist complex and diverse mechanisms.

**Table 1:** Anti-Hu encephalitis cases with EPC in previous literature, and our case.

Study	Age (years), Sex	Clinical presentations	Itcal EEG	MRI	anti-Hu antibody in serum	Neoplasm	Treatment	Prognosis
Shavit et al. (11)	56, F	dysesthesia of left trunk,left hemiparesis, myoclonus of left extremities	PDs in the right parietal area	Nonenhancing lesion in right postcentral area in T2- weighted	+	SCLC	IVIG, plasmapheresis, CTX	EPC disappeared, left hemiparesis improved, but recurred after 2 years
	54, M	generalized tonic-clonic seizure, focal-clonic seizures of right face, dysarthria, central facial paresis	PDs in the left parasagittal frontoparietal area	Normal	+	no tumor identified	not mentioned	died due to aspiration pneumonia after 30 days
	43, M	lingual myoclonus, focal-clonic seizures of left hand and face	PDs in the right frontoparietal area	Nonenhancing lesion in right Rolandic area	+	small gastric carcinoma	CZP, carboplatin, etopiside	symptoms greatly improved
Cabreira et al. (3)	15, M	focal onset seizures, left hemiparesis, dysarthria	Focal slowing and PDs in the right frontal region	Nonenhancingin right middle frontal gyrus	+	mediastinal seminoma	VPA, LCS, PER, CLB, FBM, eslicarbazepine, PB, CZP, ketogenic diet, steroids, IVIG, AZP, RTX, plasmapheresis, lesion resection, hemispherotomy	symptoms deteriorate 9 months after a transient initial improvement and seizure freedom after hemispherotomy
Mut et al. (12)	46, F	myoclonic right arm jerking, ataxia, confusion, vertical diplopia, memory deficits	Diffuse background slowing	Nonenhancing lesion in the left temporal, right frontal, left parietal areas	+ (1:30,720)	SCLC	PHT, dexamethasone, prednisone, radiotherapy, cisplatin, irinotecan, etoposide	symptoms recurred after 18 months asymptomatic interval
Porta-Etessam et al. (13)	66, M	clonic twitching of left face muscles, memory loss, personality changes	PDs in the right frontal lobe	Nonenhancing lesions in right frontal and medial bitemporal lobes	+	SCLC	PHT, CBZ, VPA	died of respiratory failure
Kinirons et al. (14)	48, F	generalized tonic-clonic convulsions, myoclonic jerks of the tongue and palate, deficits of attention and verbal memory, dysarthria, ataxia	Background slowing with PDs in the right anterior temporal region	High signal intensity in the mesial temporal structures bilaterally	+	SCLC	PHT, CBZ, steroids, chemotherapy, radiotherapy	died after 6 months
Viola et al. (15)	66, F	Head and eye right deviation and right facio-brachial myoclonia, behavioral changes	Continuous epileptiform discharges in the left temporal region	Multifocal bilateral cortical-subcortical non enhancing lesions on T2-weighted sequences	+	SCLC	LEV, LCS, methylprednisolone, plasmapheresis, carboplatin, etoposide	mild cognitive deficits with no neurological deficits or seizures
Sweeney et al. (16)	11, F	Focal jerking of the left face and arm, ataxia, opsoclonus myoclonus	Focal slowing in the left frontal and right posterior head regions	Hyperintense T2/FLAIR signal in the right mesial temporal lobe and left subinsular region	+ (1:7,680)	ganglioneuroblastoma	OXC, LEV, PHT, CLB, prednisone, IVIG, CTX, doxorubicin,vincristine, RTX	symptoms had no resolution
Our case 2025	3, M	Ataxia, focal jerking of the right fingers and arm, paralysis of right upper limb	Continuing rhythmic discharges in the left fronto-central-parietal regions	Hyperintensity signal on T2/FLAIR in the left frontal region	+ (1:1,000)	neuroblastoma	carboplatin, CTX, doxorubicin, etoposide, OXC, VPA, CZP, IVIG, methylprednisolone, prednisone, MMF	ataxia alleviated, the paralys is fully recovered and EPC disappeared, but recurred after 1 month

PDs, periodic discharges; CTX, cyclophosphamide; LCS, lacosamide; PER, perampanel; CLB, clobazam; FBM, felbamate; AZP, azathioprine; RTX, rituximab; PB, phenobarbital; LEV, levetiracetam; PHT, phenytoin; CBZ, carmazepine.

There is no definitive treatment for anti-Hu encephalitis now. First line therapy combines the treatment of the underlying malignancy and immunosuppressive therapies to taper the inflammatory process. Some patients could benefit from IVIG, corticosteroids, and plasma exchange. Anti-Hu antibodies is not thought to be directly toxic but has been associated with the presence of Hu-specific T cells (19). Drugs mainly acting on all lymphocytes or T cells may be useful, such as MMF, CTX, AZP, tacrolimus, and cyclosporine A (20). Antiseizure medications (ASMs) are usually administered but they usually fail to control the seizures (18). Furthermore, as the abnormal immune response could further affect other areas of the brain, resective surgery alone may not be sufficient to control the progression of the disease and may not be curative. Irreversible damage to neurons may occur at an early stage, so the prognosis of this disease is speculated to be poor (21). Notably, there was a tendency of anti-Hu encephalitis associated with EPC to recur (12). And this suggested the patients should probably be kept on immunosuppressants and have close follow-up.

## 4 Conclusion

This case broadens the spectrum of anti-Hu encephalitis and EPC may represent a rare but underrecognized electroclinical feature of anti-Hu encephalitis. We highlight the importance of considering anti-Hu encephalitis in patients presenting with new-onset EPC. The heterogeneity in clinical symptoms, EEG and imaging manifestations in patients may indicate the existence of complex and diverse mechanisms. ASMs and surgery often fail to control the progression of the disease. Immunotherapy may benefit some patients.

## Data Availability

The raw data supporting the conclusions of this article will be made available by the authors, without undue reservation.
